# Bulletin of the Regional Medical Chamber as a forum for ethical discourse in psychiatry and sexology

**DOI:** 10.1192/j.eurpsy.2022.1532

**Published:** 2022-09-01

**Authors:** W. Kosmowski

**Affiliations:** Nicolaus Copernicus University, Department Of Psychiatry, Bydgoszcz, Poland

**Keywords:** ethics, debate, multidisciplinary, ethical code

## Abstract

**Introduction:**

Since last year there has been a lively ethical discussion in Poland about the influence of religion and new cultural currents on medical ethics. There are many ways to work towards increasing ethical sensitivity in education of mental health care professionals.

**Objectives:**

This paper presents the ethical discourse in the bulletin “Primum” of Bydgoszcz Medical Chamber (24 chambers in Poland associate doctors and dentists): www.bil.org.pl/primum - English translations: www.dropbox.com/s/xthu7wyt56ecjwp/Primum_translations.docx?dl=0.

**Methods:**

All texts dealing with issues described were collected and divided into three groups: promoting new currents of thought, faithful to tradition, others. Presented views were analyzed basing on Polish Code of Medical Ethics (nil.org.pl/uploaded_images/1574857770_kodeks-etyki-lekarskiej.pdf) and compared with dominating philosophical schools.

**Results:**

A total of 33 articles were published: 20 presenting new approach to medical ethics, supported by the Editorial Board (72% of the total), 7 embedded in traditional values (22%), 6 without a clear stand or denying the discourse on ethical issues (6%). Articles presented philosophical views (personalism, virtue ethics, utilitarianism, constructionism), discussed ethical standards, actions contrary to the dignity of medical profession, value of human life, compliance of arguments with medical knowledge, principles of dealing with patients in terminal states, the duty of care for the pregnant woman and her child.

**Conclusions:**

All texts show dilemmas in our environment, reflect views in Polish society and in ethical discourse around the world. Thanks to them, readers familiarize themselves with the contemporary ethical debate and form their own opinions; also they are encouraged to reach for the indicated sources and their own research.

**Disclosure:**

No significant relationships.

